# Electrochemical Screening and Evaluation of Lamiaceae Plant Species from South Africa with Potential Tyrosinase Activity

**DOI:** 10.3390/s19051035

**Published:** 2019-02-28

**Authors:** Ninon G.E.R. Etsassala, Tesfaye Waryo, Olugbenga K. Popoola, Adewale O. Adeloye, Emmanuel I. Iwuoha, Ahmed A. Hussein

**Affiliations:** 1 Chemistry Department, University of the Western Cape, Private Bag X17, Bellville 7535, South Africa; 3415216@myuwc.ac.za (N.G.E.R.E.); twaryo@uwc.ac.za (T.W.); 2 Department of Chemistry, Cape Peninsula University of Technology, Private Bag X6, Bellville 7535, South Africa; olugbengapopoola@ymail.com (O.K.P.); aadeloye@cput.ac.za (A.O.A); mohammedam@cput.ac.za (A.H.)

**Keywords:** screening, Lamiaceae, extracts, tyrosinase inhibitors, cyclic voltammetry, cosmetics

## Abstract

South Africa is a country with a wide variety of plants that may contain excellent anti-tyrosinase inhibitors. With wide applications in cosmetics, pharmaceuticals and food products, tyrosinase inhibitors have received very special attention in the recent past as a way of preventing the overproduction of melanin in epidermal layers which often over time brings detrimental effects on human skin. In this present study, a fast screening method using a cyclic voltammetry technique was applied in the evaluation of methanolic extracts of twenty-five species of plants from the Lamiaceae family for anti-tyrosinase activity. Among these plants, those that showed a fast current inhibition rate at a minimum concentration when compared to a kojic acid standard were classified as having the greatest anti-tyrosinase activity. These include *Salvia chamelaeagnea*, *S. dolomitica*, *Plectranthus ecklonii*, *P. namaensis,* and *P. zuluensis*. The results presented herein focused in particular on providng firsthand information for further extensive research and exploration of natural product materials with anti-tyrosinase activity from South African flora for use in cosmetics, skin care and medicinal treatments.

## 1. Introduction

Tyrosinase is a copper-containing enzyme present in animal, plant and human tissues. Two distinct reactions in particular involving molecular oxygen (O_2_) that are known to be catalyzed by this enzyme include hydroxylation of tyrosine to 3,4-dihydroxyphenylalamine (DOPA) and oxidation of DOPA to DOPA-quinone by the actions of monophenolase and diphenolase, respectively [1,2,3]. Tyrosinase has been considered as the rate-limiting enzyme in the control and production of the pigment melanin (a dark macromolecule produced during the process of melanogenesis) [4]. The enzyme plays significant role as a photo-protective agent against the harmful effects of ultraviolet (UV) radiation caused by the absorption of UV light and reactive oxygen species [5]. In addition, as a result of these effects, the skin appearance in humans is determined. Prolonged exposure of human skin to UV radiation leads to over-accumulation of free radicals in the body which has been a factor implicated in the stimulation of skin degenerative tyrosinase enzymes as a result of undesirable skin hyperpigmentation formation, including premature skin aging [6]. Significant efforts have been recorded in the search for active skin depigmenting agents from synthetic [7,8] and natural product sources [9,10,11]. Due to persistent occurrence of these unpleasant changes in the structural integrity and physiological function of the skin [12,13], numerous ingredients have been introduced as skin whitening agents in cosmetic formulations, including hydroquinone, kojic acid, arbutin, and azelaic acid, which are readily available in the market [14]. The effectiveness of these products is a challenge based on their adverse side effects, poor skin penetration and low environmental stability [15]. There is therefore a great need to search for new active and better natural tyrosinase inhibitors for use in modifying skin pigmentation which will have less side effects, wide acceptability and a superior safety margin when compared to synthetic products [16].

Different methods including spectrophotometric assays, TLC bioautographic assays, high performance liquid chromatography, electrophoresis, isotope assays, enzyme-linked immunosorbent assays and electrochemical techniques have been reported for both the qualitative and quantitative measurement of tyrosinase activity [6,17]. Among these assay methods, electrochemical measurements are affordable, reliable and robust tools for measuring the antioxidant capacity of plant extracts [18] and have also been applied in cosmetic formulations and the detection of phenolic compounds in extracts and wines [19].

Lamiaceae plant species are widely distributed among the South African flora and comprise about 255 species which are assigned to 35 genera [20]. These plant species have been used in traditional medicine to treat different ailments and diseases. The family is a rich source of phenolic compounds such as flavonoids and phenolic acids. Some of the species contain diverse other phytochemicals, including abietane diterpenes [21]. Some of these phytochemical compounds are expected to play important roles in the control of undesirable skin conditions either by an antioxidant activity mechanism or inhibition of the tyrosinase enzyme [22]. Several reports have shown that natural phenolic compounds have wide applicability in the formulation of cosmetic products as well as potential anti-tyrosinase agents. This study specifically focused on the use of a fast cyclic voltammetry method in the preliminary screening of the methanolic extracts of 25 plant species from the Lamiaceae family indigenous to South Africa for their anti-tyrosinase activity.

## 2. Materials and Methods

### 2.1. Plant Materials

The plant materials used in this study were collected from different localities in the Western Cape Province of South Africa which included Kirstenbosch Garden Centre, Nursery of the Cape Flats Nature Reserve, Cape Flats Nature Reserve and Hantam National Botanical Garden, Nieuwoudtville. Identification of the plants was carried out at the Compton Herbarium, Kirstenbosch. Voucher specimens were deposited at the Compton Herbarium (NBG), Kirstenbosch.

### 2.2. Preparation of Plant Extracts

The aerial part of each fresh plant materials was macerated and extracted in methanol for 24 h at room temperature (25 °C). The methanolic extract of each plant was filtered exhaustively and then evaporated to dryness under reduced pressure at 40 °C. The extracts were kept in an airtight glass sample vials under cold conditions (−5 °C) for further use.

### 2.3. Chemicals and Reagents

Mushroom tyrosinase (EC 1.14.18.1) 5771 Units/mg, L-tyrosine and kojic acid were purchased from Sigma Aldrich (Cape Town, South Africa). Methanol (MeOH) and dimethyl sulfoxide (DMSO) were supplied by Merck (Cape Town, South Africa).

### 2.4. Apparatus

The cyclic voltammetry was performed using a BASI EPSILON system (Bioanalytical systems, West Lafayette, Indiana, USA) as potentiostat. Three electrodes were used, which included a glass carbon electrode (GCE) as the working electrode, a platinum (Pt) wire electrode as the counter electrode and an auxiliary electrode Ag/AgCl as reference electrode. The electrodes’ surface was polished with alumina paste (1, 0.3 and 0.05 µM) on a Buehler felt pad, followed by sonication in ethanol and finally rinsed with excess Millipore water prior to use. All the electrochemical experiments were performed in PBS as supporting electrolyte. All experiments were performed in triplicate at room temperature.

### 2.5. Cyclic Voltammetry Measurement

Cyclic voltammetry (CV) is a type of potentiodynamic electrochemical measurement technique which consists of an electrode with an applied voltage swept between two values, and a current measured between a working and counter electrode which also serve as a function of the potential [23]. CV was used to evaluate the tyrosinase inhibitory activity of the plant extracts by measuring the inhibition current at multiple time points. These data are plotted as current (I) versus applied potential (*E*) and recorded at two different scan rates of 50 mV/s and 25 mV/s within the potential window range −1300 to 1300 mV and −200 to 200 mV. A stock solution of 10 mg/mL of methanolic extracts of each plant and kojic acid were prepared in DMSO. Tyrosinase and L-tyrosine in diluted phosphate buffer solutions were prepared to serve as working concentration. All the experiments were carried out at room temperature. To a cell containing 10 mL of 50 mM PBS at pH 6.5, a fixed volume of extracts and tyrosinase enzyme were added (700 and 300 µL) respectively, followed by addition of specific volume of L-tyrosine (~1100 µL). To understand the bioactivity, a control set- up (positive or negative) of plant extracts was replaced by kojic acid and/or DMSO, which allows the cyclic voltammetry experimental measurements of each addition to be recorded in triplicates.

## 3. Results and Discussion

### Cyclic Voltammetry Measurement

**Figure 1:**
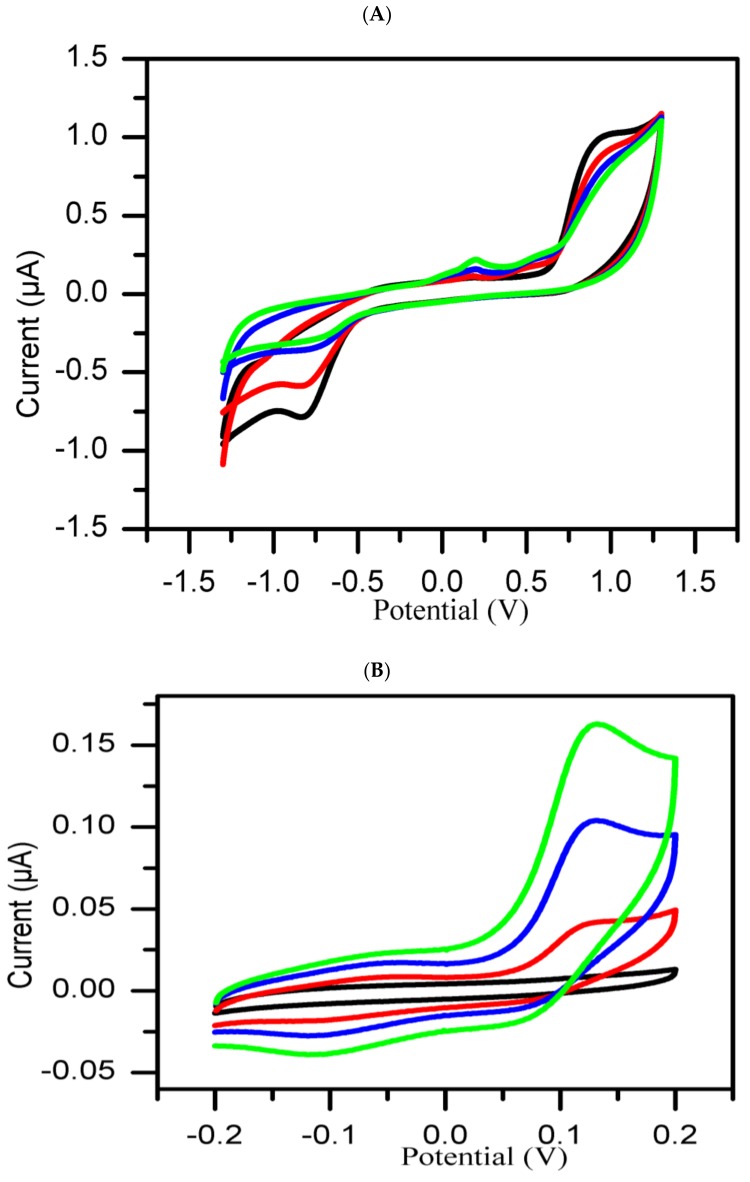
Overlay of CVs of mixture of tyrosine and tyrosinase in PBS (pH 6.5) recorded at 50 mV/s (**A**) and 25 mV/s (**B**) at different reaction durations: 0 min (Black curve), 10 min (red curve), 20 min (blue curve), and 30 min (green curve).

It is well known that tyrosinase catalyzes the oxidation process of L-tyrosine to dihydroxyphenylalamine (L-DOPA) and from L-DOPA to dopaquinone [24]. In this experiment, a protocol was designed to use cyclic voltammetry to monitor the enzymatic oxidation of L-tyrosine in the presence of molecular oxygen catalyzed by tyrosinase. As shown in Figure 1A, broad-scan range set of CVs at −1300 to 1300 mV at 50 mV/s show interesting oxidation peak potential at 890 mV. This peak potential was attributed to the oxidation of tyrosine [25], while a corresponding reduction peak potential was observed at −825 mV. Another oxidation peak potential observed at a potential range of −200 to 200 mV evolved only gradually by the increase in peak current with reaction time. We attribute this to the electron transfer reaction involving dopaquinone, a product of the enzymatic reaction which possibly accumulates in the reaction medium in relation to reaction time [26]. As shown in Figure 1B, this peak was confirmed to exist at about 131 mV and was found to be independent of the other peaks as when compared to another set of CV studies carried out in a narrow scan range of −200 to 200 mV at 25 mV/s. The peaks now referred to as the inhibition peak and its peak current as inhibition current (I_inh_) are expected to be affected by the presence of tyrosinase inhibitory agents.

**Figure 2:**
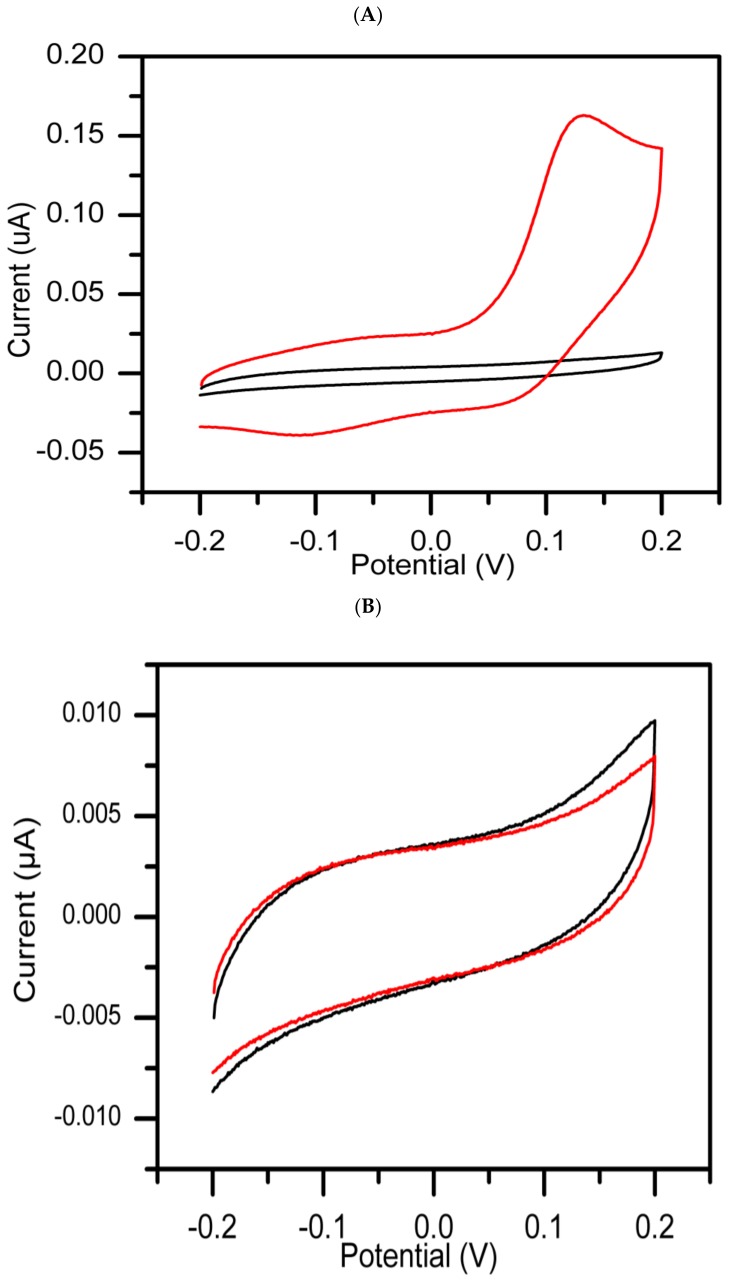
CVs recorded at 25 mV/s for the reaction mixtures of negative control (**A**) and positive control (**B**) experiments in the presence of 50 mM PBS (pH 6.5), after consecutive addition of DMSO, tyrosinase and tyrosine. The CV recorded at 0 min (black curve) and at 30 min (red curve), respectively.

The inhibition peak for the same reaction mixture was studied in the presence of kojic acid, a standard tyrosinase inhibitor [27], to serve as positive control (PC) and DMSO as negative control (NC). As shown in Figure 2A, an increase in oxidation peak was found to be directly correspond to increase in oxidation current from 8.99 × 10^−3^ to 9.60 × 10^−3^ µA at 131 mV. This could be explained in terms of tyrosine oxidation being catalyzed by tyrosinase enzyme. In Figure 2B, no redox peak was observed, a decrease in inhibition current which almost parallel to the background level with time, indicates that the activity of tyrosinase was inhibited by kojic acid which is a known standard chelator of the copper ion on the active site of the enzyme [14]. These results are in agreement with previously described work [17], and the proposed method should be applicable for screening tyrosinase inhibitors from natural sources.

Based on the cyclic voltammogram experimental results, the inhibitory activity potential of methanolic extract of each plant against tyrosinase enzyme was classified as either positive or negative compared to the CVs recorded for the standard kojic acid as positive control or DMSO as negative control. Accordingly, inhibitory behavior is termed “positive” when the inhibition current (I_inh_) decreases with time and as “negative” with (I_inh_) increases with time. The inhibitory activity of each extract using the CV was monitored and recorded based on time difference between 0–30 min, and the addition of substrate to a mixture of extract and enzyme in buffer. Extracts are classified into two groups: Group 1 (active extracts) and Group 2 (inactive extracts):

*Group 1:*  The extracts in this group referred to as active, showed inhibitory activity potential against tyrosinase enzyme compared to standard kojic acid used as positive control, which gave a significant decrease in inhibition current with time.
*Group 2:*  The extracts in this group are classified as inactive on the basis of their poor inhibitory activity against tyrosinase enzyme as compared to the standard negative control (DMSO). The inhibition current significantly increasesd with time.

**Figure 3:**
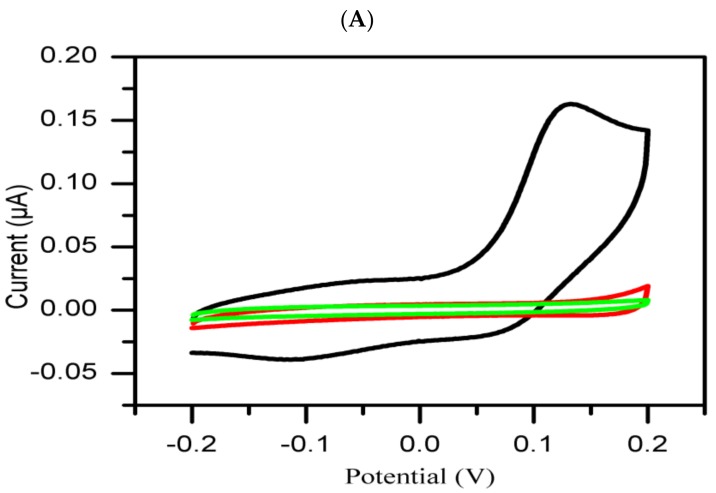

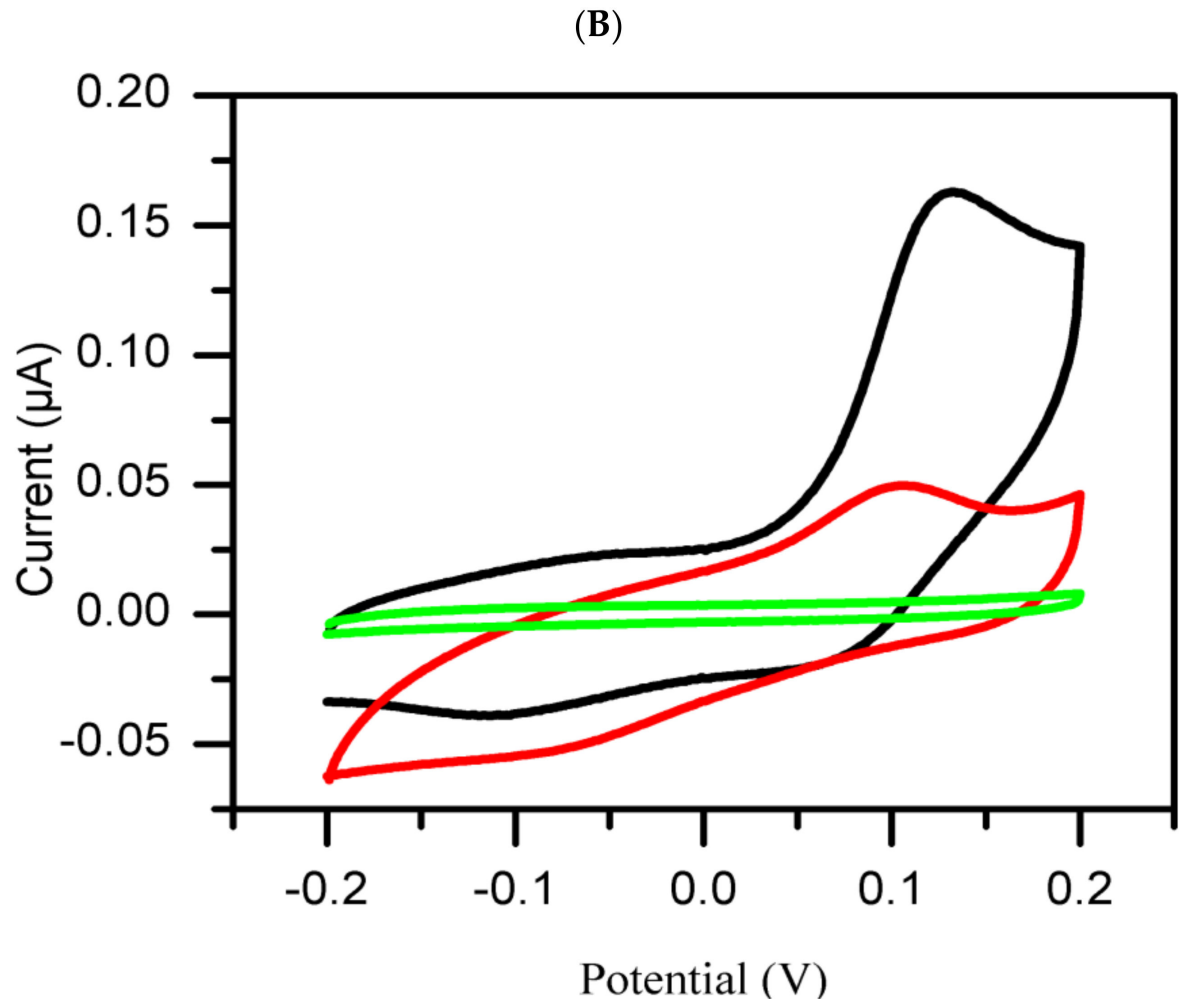
Comparative analysis of representative CVs of each group: Inactive extract (**A**, red curve) and active extract (**B**, red curve) with negative control (black curve) and positive control (green curve).

As mentioned earlier, representative CVs of the extracts per group at 0 min and 30 min after the addition of tyrosinase and tyrosine are shown and compared to the corresponding CVs obtained for the positive and negative controls. The histograms of the groups 1 and 2 obtained by measuring the inhibition current of each extract and controls at a potential of 131 mV and in the intervals of 0–30 min after adding tyrosinase and tyrosine to the extract(s) or control(s) are shown in Figure 3A,B.

The results above demonstrate the tyrosinase inhibitory activity of kojic acid obtained by measuring the inhibition current at multiple time points using a cyclic voltammetry method. It has also been found that during the screening of tyrosinase inhibitors by cyclic voltammetry, the current increases with the increase of tyrosinase concentration in the absence of inhibitor, while the inhibition current decreased and almost dropped to the background level when the kojic acid concentration was increased, an indication that the activity of tyrosinase was significantly inhibited by kojic acid [17]. Although various chemical scaffolds have been discovered in the treatment of hyperpigmentation and other related skin disorders, the presence of a 3-hydroxyl and 4-ketone group in the kojic acid structure may play very important role as a coordinating site for copper atom oxidase in tyrosinase inhibition [28]. 

**Figure 4:**
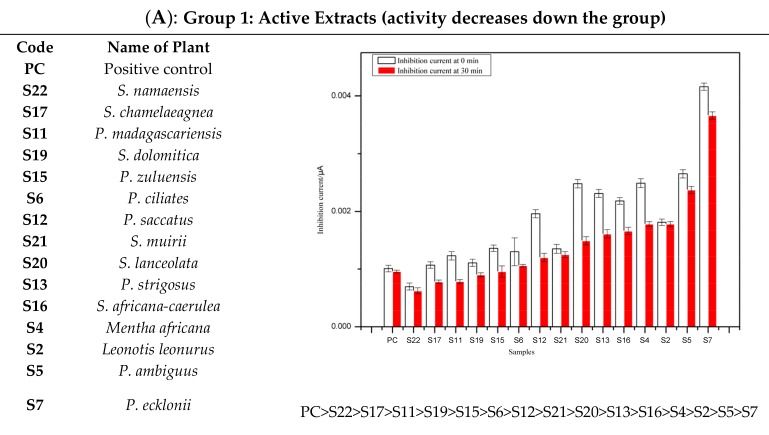

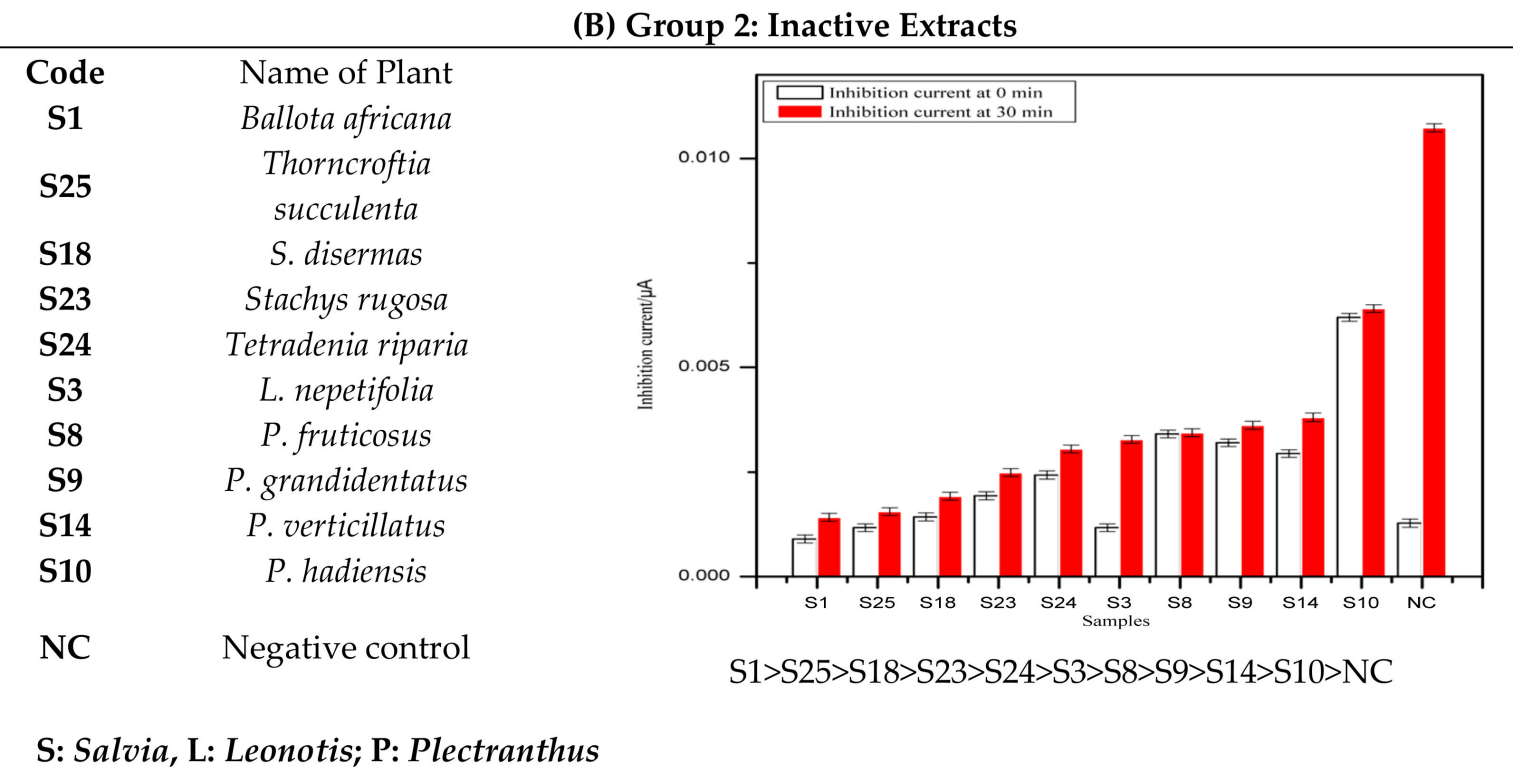
Illustration of the effect of groups on inhibition of tyrosinase enzyme activity. (**A**) Group 1: Active Extracts; (**B**) Group 2: Inactive Extracts. The histograms are expressed as mean ±SEM for n = 3.

As shown in Figure 3A and Figure 4A, all active extracts when compared to kojic acid (positive control) are found to decrease the inhibition current with time. It is believed that the rapid interaction of the inherent phytochemicals present in the extracts might be related to the degree of chelation sites responsible for the inhibitory activity of the tyrosinase enzyme.

A preliminary phytochemical investigation previously carried out in our research group (results reported elsewhere) on the methanolic extracts of the 25 plants revealed positive tests for presence of polyphenolic and terpenoids compounds. The varied levels of decreased inhibition current may therefore be ascribed to the presence and/or extent of polyphenolic constituents present in the extracts which have tendency to form chelate-complexation with enhanced inhibitory activity [16]. As shown in Figure 3B and Figure 4B, no tyrosinase inhibitory activity was observed in some extracts as well as the DMSO (negative control) based on increased inhibition current with time. The apparent tyrosinase enzyme inactivity may be related to the types of phytochemical constituents and/or structures present in such plant species. We envisaged their weak reaction with tyrosinase enzyme, thus enhancing production of melanin, and the overproduction of melanin [29].

## 4. Conclusions

Melanin overproduction has been implicated as the main cause of many disorders such as hyperpigmentation, melasma, and age spots in humans. Tyrosinase is directly linked to the biogenesis of melanin. In recent years, various analytical methods have been used in both the qualitative and quantitative measurement of tyrosinase activity. This study reports an investigation using a cyclic voltammetry method of analysis establishing the tyrosinase inhibitory activities of methanolic extracts of 25 plant species belonging to the Lamiaceae family indigenous to South Africa. Electrochemical screening through the use of cyclic voltammetry is an affordable, rapid and robust analytical tool used in the measuring of antioxidant capacity in plant extracts, detection of tyrosinase inhibitors, and in cosmetic formulations when compared to other methods such as TLC bioautography, isotope and spectrophotometric assay techniques. The method, in addition to its role in research, if properly used would be suited and reliable for collecting quality control data and extraction procedure optimization. The results obtained from this assay in addition to the preliminary phytochemical investigation show that further work to isolate the bioactive compounds responsible for the tyrosinase inhibitory activities of the plants extracts will aid in the establishment of mechanism of action.
